# Supplementary material for: Development of a computer-aided design software for the quantitative evaluation of aesthetic damage
Source: PLoS One. 2019 Dec 18;14(12):e0226322. doi: 10.1371/journal.pone.0226322 (PMC6919621; doi:10.1371/journal.pone.0226322)
Supplement: S1 Code site — 1. (ZIP) [file pone.0226322.s001.zip › [EESC_jr][Projeto]Relatorio_07_21/vendors/select2/docs/_includes/options/compatibility/text-input.html]

## Compatibility with `<input type="text" />`

Deprecated in Select2 4.0.
It is now encouraged to use the `<select>` tag instead.

In past versions of Select2, a `<select>` element could
only be used with a limited subset of options. An
`<input type="hidden" />` was required instead, which did
not allow for a graceful fallback for users who did not have JavaScript
enabled. Select2 now supports the `<select>` element for
all options, so it is no longer required to use `<input />`
elements with Select2.

Adapter
:   `DataAdapter`

Decorator
:   `InputData`
